# Allocation of Resources to Growth and Spore Production in a Fern *Ophioglossum vulgatum* L.: Effects of Mowing and Simulated Herbivory

**DOI:** 10.1002/ece3.71555

**Published:** 2025-06-13

**Authors:** Natalia Jędrzejczak, Paweł Olejniczak, Zbigniew Celka

**Affiliations:** ^1^ Department of Plant Ecology and Environmental Protection Institute of Environmental Biology, Faculty of Biology, Adam Mickiewicz University Poznań Poland; ^2^ Institute of Nature Conservation Polish Academy of Sciences Kraków Poland; ^3^ Department of Systematic and Environmental Botany Institute of Environmental Biology, Faculty of Biology, Adam Mickiewicz University Poznań Poland

**Keywords:** adder's‐tongue fern, competition, fern ecology, herbivore, reproduction, resource allocation

## Abstract

Natural selection drives how organisms allocate resources among competing demands such as growth, reproduction, and survival. In ferns, where reproductive and vegetative organs share developmental pathways, these trade‐offs may be particularly strong under environmental disturbance. This study investigates how the rare fern *Ophioglossum vulgatum* allocates resources between vegetative growth and reproduction in response to vegetation removal (mowing) and simulated herbivory (clipping). A field experiment was conducted in wet meadow and peatland habitats in central Poland using a factorial design. Four treatments were applied: control, clipping, mowing, and clipping combined with mowing. Across 10 transects, 533 ramets were marked and monitored. Biometric measurements included leaf blade area, sporophore length, number of sporangia, and plant height. Data were analyzed using two‐way ANOVA with clipping and mowing as fixed factors. Key results: Mowing significantly reduced plant height and leaf blade area, but only in unclipped plants (significant clipping × mowing interaction). Clipping alone, simulating herbivory, had no significant direct impact on any of the measured traits, although its interaction with mowing revealed important effects. Control plots exhibited the greatest allocation to reproduction, with larger sporophores and more sporangia. All treatments reduced reproductive output compared to controls, while vegetative performance remained stable or increased. Ramet abundance increased across all treatments, suggesting resilience through clonal propagation. These findings suggest that *O. vulgatum* exhibits trait‐specific and context‐dependent responses to disturbance. Reproductive traits are more sensitive than vegetative growth, and their suppression under mowing may limit reproductive success in managed habitats. Conservation strategies should account for both short‐term physiological responses and long‐term demographic processes. Management practices, particularly mowing, should be carefully timed and scaled to avoid unintended negative effects on reproduction in rare ferns such as *O. vulgatum* .

## Graphical abstract

The study explores how the rare fern *Ophioglossum vulgatum* allocates resources between growth and reproduction under mowing and simulated herbivory. The results reveal that mowing promotes photosynthetic growth at the expense of spore production, while herbivory shows no significant impact, highlighting the fern's resilience. The findings underline the need for tailored conservation practices to balance growth and reproduction, ensuring the species persistence in its habitat.

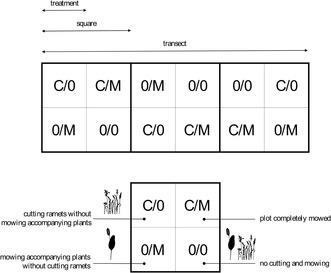

## 1 Introduction

Every organism allocates its resources among essential life processes, such as growth, reproduction, and defense (Stearns 1992). The distribution of these resources varies among organisms, depending on species‐specific biological traits and the living conditions of the individual. Since resources are limited, organisms face allocation dilemmas, such as “deciding” the optimal timing for the initiation of reproduction. Any “decisions” made by organisms entail measurable costs.

The allocation of resources in seed plants has been a subject of research since the 1970s (e.g., van Noordwijk and De Jong 1986; Doust 1989; Boersma 1995; Bazzaz and Grace 1997; Miyazaki et al. 2002; Averill 2014; Mironchenko and Kozłowski 2014; Wiernasz and Cole 2018). Studies on resource allocation in plants have elucidated the causes of life‐history trait variability (e.g., offspring number per reproductive event, body size, maximum lifespan) at both interspecific (e.g., Roff et al. 2006; Stearns 1992) and interpopulational levels (e.g., Reznick et al. 1997; Czarnołęski et al. 2005; Bañuelos and Obeso 2004; Czarnołęski et al. 2013; Daimon et al. 2014).

In contrast, analyses of resource allocation to life processes in spore‐bearing plants are rare, particularly those based on field experiments (Rünk and Zobel 2007; Coelho et al. 2021; Li et al. 2023). Understanding resource allocation mechanisms in spore‐bearing plants, particularly ferns, is of special interest due to their evolutionary history, which has led to a complex morphological structure. During the evolution of the family Ophioglossaceae, the number of leaves was reduced to a single one, divided into a sterile and a fertile part, distinguishing them from other ferns. In contrast to Ophioglossaceae, ferns from the order Polypodiales, which constitute over 80% of modern fern species, exhibit greater morphological diversity and more complex leaf structures. Ophioglossales is considered one of the most ancient lineages among ferns, with molecular phylogenetic analyses estimating its divergence from other fern clades at approximately 250–280 million years ago (Mya), during the late Paleozoic to early Mesozoic era (Pryer et al. 2004; Zhang et al. 2020). The family currently includes 112 species worldwide, classified into 10 genera according to the Pteridophyte Phylogeny Group I (PPGI 2016). This classification distinguishes genera such as *Ophioglossum* s.l. and *Botrychium* s.l. as broader taxa that encompass previously recognized subgroups. The structures responsible for spore reproduction develop at an early stage of leaf development. Unlike leptosporangiate ferns, where the sporangia are located on the underside of the leaf blade, in the Ophioglossaceae they are positioned at the leaf apex (Schneider et al. 2009). In genera such as *Botrychium* Sw., *Helminthostachys* Kaulf., or *Mankyua* B. Y. Sun, M. Kimthe, the spore‐bearing part is branched, while in *Ophioglossum* L., a single spike is produced (Campbell 1911; Barker and Hauk 2003).

**FIGURE 1:**
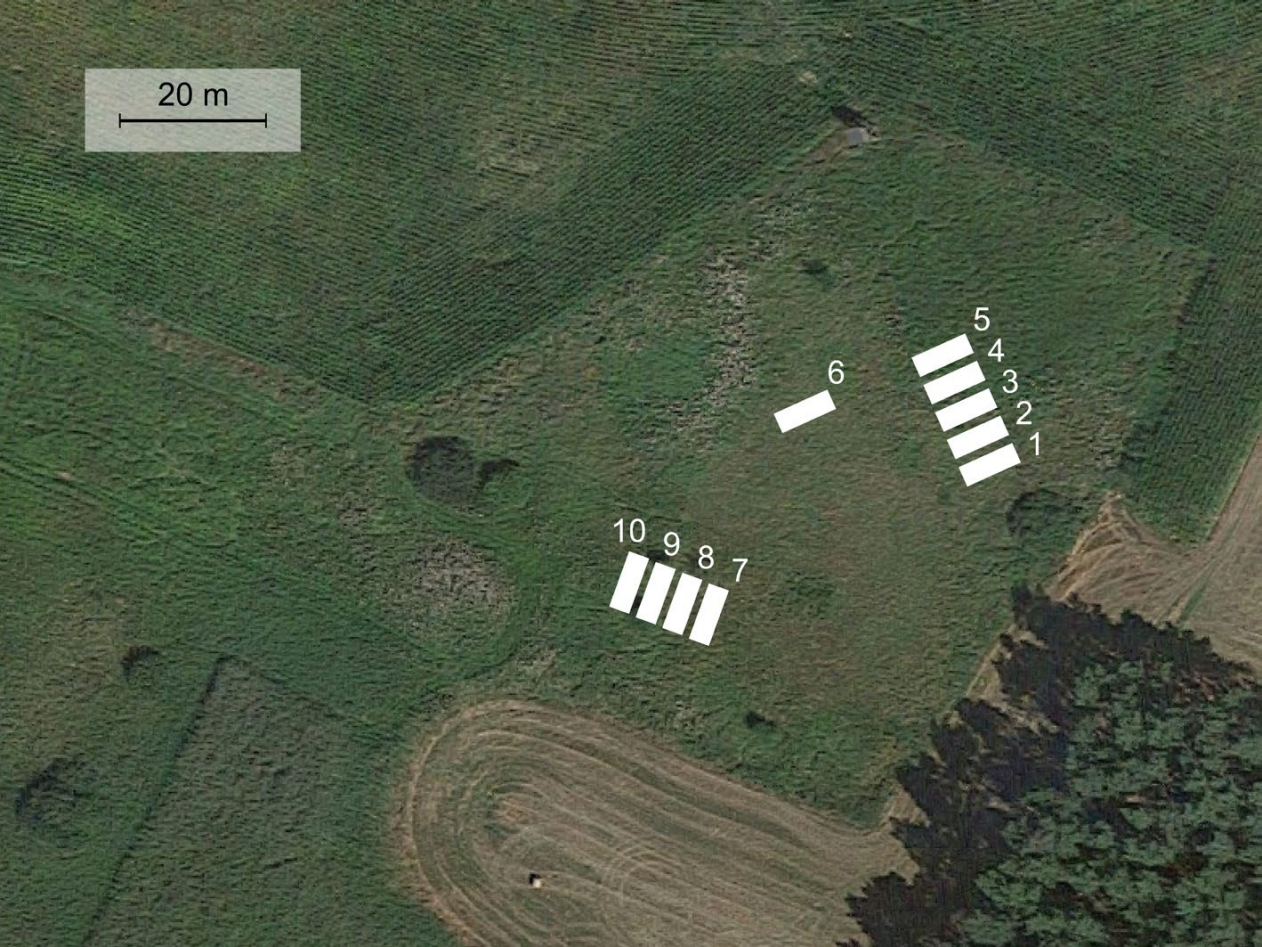
Placement of transects in the field (*Source:* Google Maps, modified).

*Ophioglossum vulgatum* is a small perennial fern, with sterile blades typically ranging from 2.5 to 10 cm in length and 1 to 4 cm in width, and fertile spikes reaching up to 30 cm (Figure 1). Sporophytes usually emerge in early summer, with spore maturation occurring in late June or July. Individual leaves are short‐lived, persisting for approximately 2–3 months. Following senescence, the plants remain underground and leafless for the remainder of the year, and in some cases may stay dormant for one or more growing seasons (Edwards 1982; McMaster 1994). This life history is important in the context of resource allocation in *O. vulgatum* , which produces only a single leaf per year and often suppresses spore production. The leaf consists of an undivided, oval, reticulate‐veined trophophore (the assimilative part) and a sporophore (the spore‐bearing part). The sporophyte does not emerge every year. When sporangia mature, they release large quantities of spores (Mehltreter et al. 2010). During the growing season, the sporophyte exhibits a “decision process” as a result of which a ramet develops either solely the assimilative part or both the assimilative and spore‐producing parts. However, there is always the formation of a single leaf emerging from the rhizome. In this study, the adders‐tongue fern *Ophioglossum vulgatum* L. (Ophioglossaceae) was examined. This perennial plant produces only one leaf per growing season. In certain years, individuals may remain dormant underground without producing a leaf (Johnson‐Groh 1998). This species is circumboreal, widespread in lowland and lower mountain regions in Europe (Hultén and Fries 1986; Zając and Zając 2001; Stadnicka‐Futoma and Jaźwa 2020). In Poland, *O. vulgatum* is strictly protected and requires active conservation measures, such as mowing its habitats. It is also classified as LC on the European Red List of Ferns (García Criado et al. 2017). Recent studies highlight that in spore‐bearing plants, environmental disturbances such as mowing and herbivory can significantly influence the balance between vegetative growth and reproductive investment (Coelho et al. 2021; Li et al. 2023). Moreover, Li et al. (2023) showed that while the overall resource economics spectrum in ferns may remain stable across environments, the allocation between growth and reproduction can still vary in response to local disturbance regimes. Our experiment aimed to answer two questions related to the persistence of the fern *O. vulgatum* . Firstly, how does mowing the surrounding vegetation affect resource allocation to the photosynthetic and spore‐bearing parts? This factor is particularly important for the continued active protection of this endangered species. Secondly, how does simulated herbivory impact the photosynthetic and spore‐bearing parts of *O. vulgatum* ?

## 2 Materials and Methods

### 2.1 Experimental Design

#### 2.1.1 Marking the Transects

**FIGURE 2:**
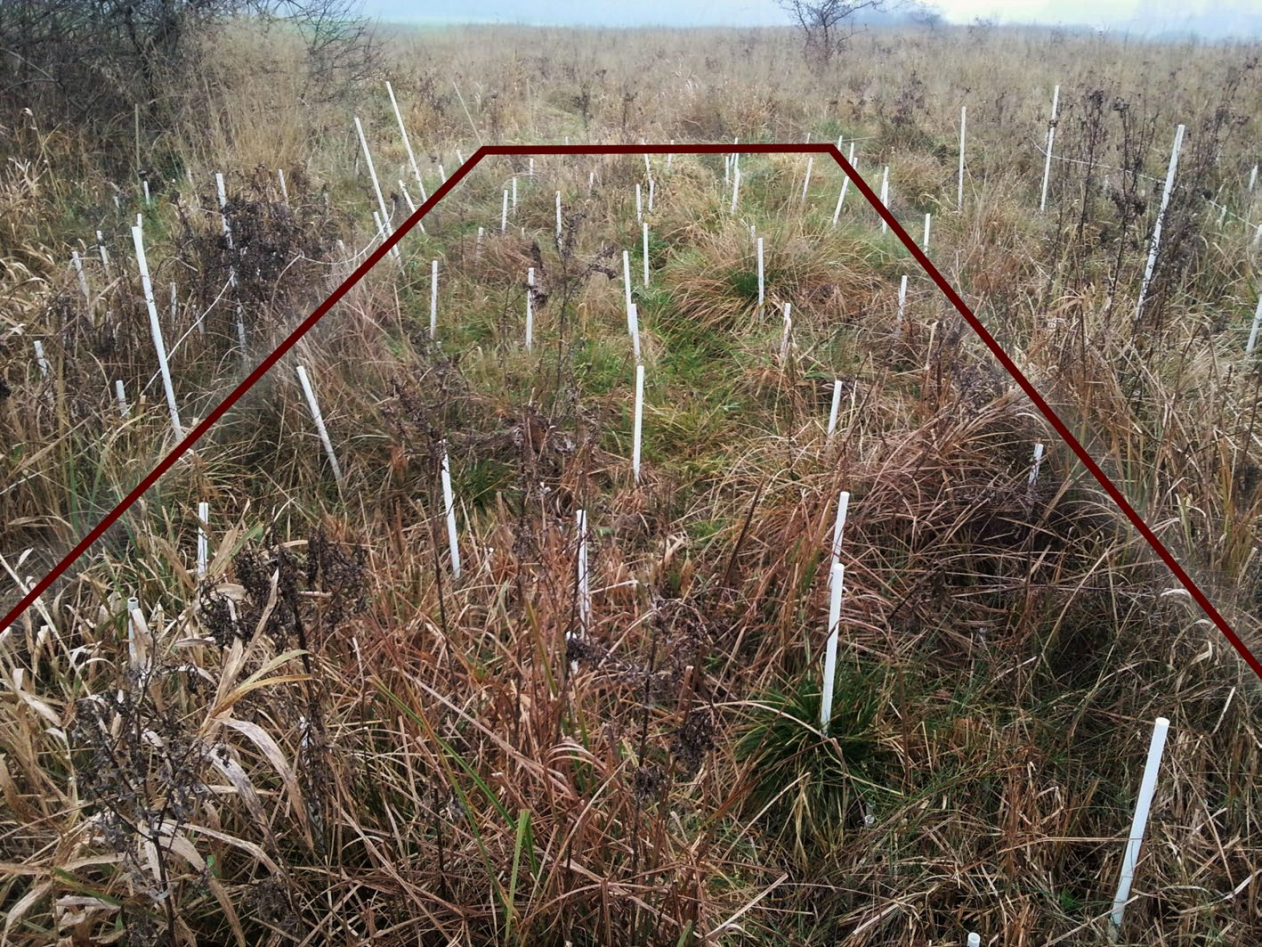
Transect marked in the field (photo by Natalia Jędrzejczak).

The study was conducted in the summer of 2014 and 2015 in northwestern Poland (Central Europe), near the village of Imielenko (52.488199°N, 17.390278°E) within a complex of meadows and wetlands. In June 2014, 10 transects were established (numbered 1–10), each 8.1 m long and 2.7 m wide. The transects covered a similar range of habitats: from wet sedge meadows (transects 1–6) to moderately moist, so‐called fresh meadows (transects 7–10) (Figure 1). Within each transect, three squares with a side length of 2.7 m were designated. The transects were marked using PVC tubes (1 m long), inserted into the corners of each square (Figure 2).

#### 2.1.2 Applied Treatments

**FIGURE 3:**
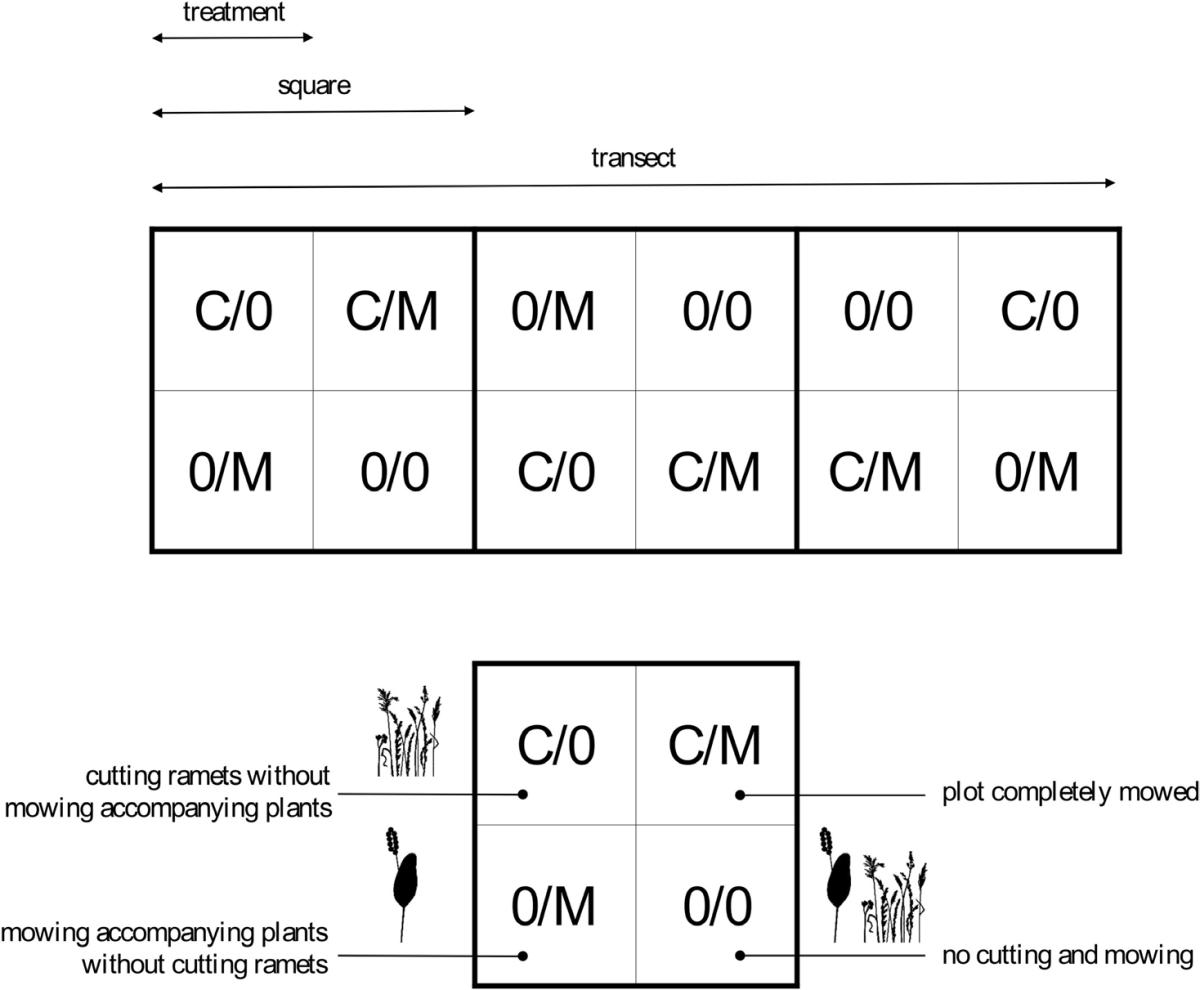
Types of treatments (with the post‐treatment conditions illustrated using symbols) and a diagram of a transect with squares and treatments.

After biometric measurements of the ramets in June–July 2014 (see below), treatments were applied in August (Figure 3). In each square within every transect, four plots measuring 0.8 m × 0.8 m were designated and subjected to one of four treatments: 0/C—clipping all fern ramets without mowing the accompanying vegetation (to simulate herbivore grazing); M/0—no clipping of fern ramets but mowing the accompanying vegetation (to simulate changes in its density); M/C—complete mowing of the entire plot; and 0/0—control: no clipping and mowing (to simulate absence of management). The vegetation was mowed with a sickle, and the fern ramets were clipped with scissors at a height of 1 cm from the petiole base. All the clipped and mowed plant parts were collected and removed from the plots.

### 2.2 Sampling and Measurements

#### 2.2.1 Size and Reproduction of Ramets

**FIGURE 4:**
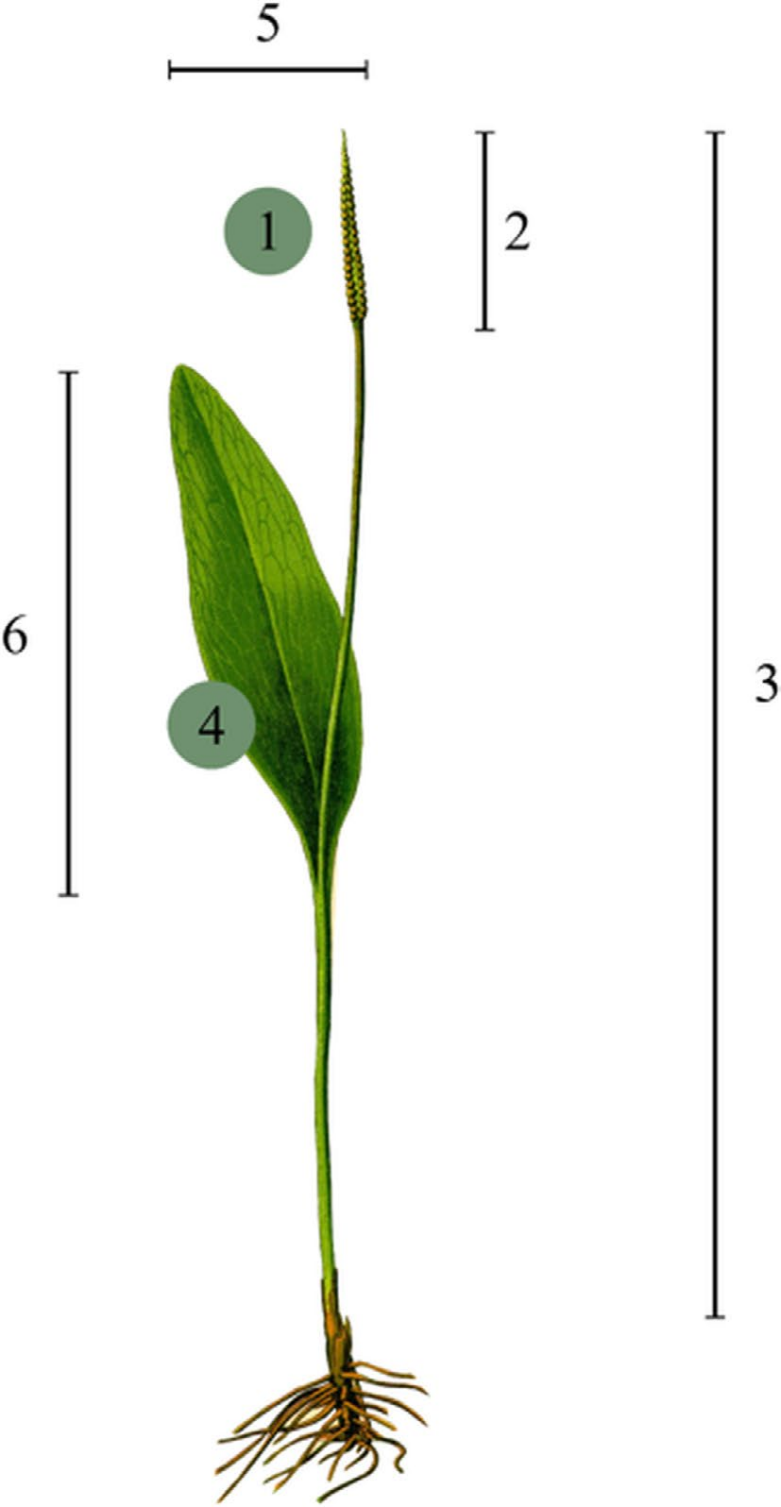
Morphological habit of *O. vulgatum*. Biometric parameters measured in the field: (1) number of sporangia, (2) length of sporophore without petiole, (3) height of whole plant measured from ground surface, (4) leaf blade area, (5) width of trophophore measured in broadest place, (6) length of trophophore.

The measure of reproduction in *O. vulgatum* was the number of ramets consisting of both trophophore and sporophore. For each ramet, the following traits were measured in June–July in 2014 and 2015: length of the sporophore excluding the petiole, width of the trophophore at its broadest point, length of the trophophore, leaf blade area (calculated using the formula for the area of an ellipse, *p = πab*, where *a* is half the length of the trophophore and *b* is half its width), height of the whole plant measured from the ground surface, and the number of sporangia (Figure 4). Ramets were measured to the nearest millimeter using a ruler when they were fully developed, that is, when the mature sporophores were present. At the same time, measurements were also taken for ramets without sporophores. A total of 533 ramets of *O. vulgatum* were measured. The leaf blade area was considered a measure of allocation to growth. The length of the sporophore excluding the petiole and the number of sporangia were treated as measures of allocation to reproduction.

### 2.3 Statistical Analysis

To examine the effects of experimental treatments, general linear models were applied with mowing and clipping as fixed factors and transect as a random factor. The biometric traits of the ramets (i.e., sporophore length, number of sporangia, plant height and log‐transformed leaf blade area) were used as dependent variables. The 2014 data were collected prior to the application of experimental treatments. No statistically significant differences among treatment groups were observed for any of the measured traits at this stage (sporophore length: *F* = 1.307, *p* = 0.276; number of sporangia: *F* = 0.925, *p* = 0.431; plant height: *F* = 2.438, *p* = 0.064; leaf blade area: *F* = 0.107, *p* = 0.956). These results confirm that the initial conditions were comparable across experimental plots. Since no significant differences among treatment groups were observed in 2014, comparisons of trait values in 2015 reflect the net effects of the treatments. Analyses were conducted separately for each trait using ANOVA. All statistical analyses were performed using IBM SPSS Statistics ver. 25.

## 3 Results

### 3.1 Effects of Clipping and Mowing the Plant Competitors on the Growth and Reproduction of *O. vulgatum*

**FIGURE 5:**
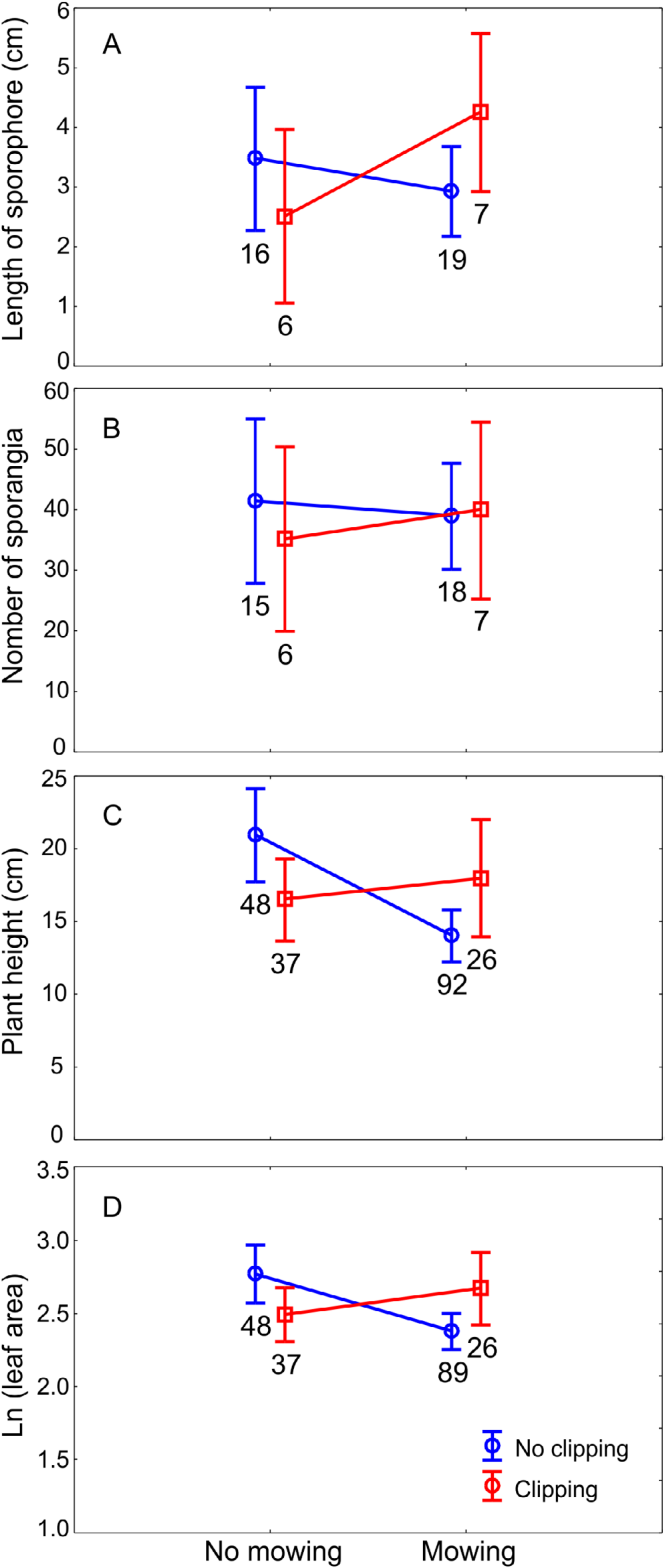
Morphological traits of *Ophioglossum vulgatum* in response to mowing of surrounding vegetation and simulated herbivory (clipping) in 2015. Panels show (A) length of the sporophore without the petiole, (B) number of sporangia, (C) plant height, and (D) leaf blade area (log‐transformed). Marginal means and standard errors are indicated. Sample sizes are shown next to each data point. Treatments: Blue line—no clipping; red line—clipping; *x*‐axis categories indicate no mowing and mowing.

We examined the effects of clipping and mowing on four morphological traits of *Ophioglossum vulgatum* , treating transect as a random factor and clipping and mowing as fixed effects (Figure 5 and Table 1). For all traits, we included the interaction term between mowing and clipping to test for combined treatment effects. For sporophore length (Figure 5A), a marginally significant clipping × mowing interaction was detected (*F* = 3.891, *p* = 0.056). In mown plots, clipped plants produced longer sporophores (mean ≈ 4.5 cm) than unclipped ones (mean ≈ 3.0 cm), while in unmown plots the trend was reversed, suggesting that the interaction may be biologically meaningful despite not reaching conventional significance. No significant effects were detected for the number of sporangia (Figure 5B). Neither mowing (*F* = 0.020, *p* = 0.888), clipping (*F* = 0.210, *p* = 0.650), nor their interaction (*F* = 0.334, *p* = 0.567) had any measurable impact. In contrast, plant height (Figure 5C) was significantly affected by the interaction of clipping and mowing (*F* = 7.401, *p* = 0.007). Among unclipped plants, mowing led to a significant reduction in plant height (*F* = 13.297, *p* < 0.001). In clipped plants, however, mowing had no significant effect (*F* = 0.312, *p* = 0.579), suggesting that mowing alone, rather than simulated herbivory, was responsible for the reduction in vertical growth. Similarly, for leaf blade area (log‐transformed; Figure 5D), a significant clipping × mowing interaction was observed (*F* = 8.062, *p* = 0.005). In the absence of clipping, mowing significantly reduced leaf area (*F* = 9.287, *p* = 0.003; *n* = 48 without mowing, *n* = 89 with mowing), whereas no significant effect of mowing was found in clipped plants (*F* = 1.021, *p* = 0.317; *n* = 37 and 26, respectively). These results indicate that mowing negatively impacts leaf area only when plants are not simultaneously subjected to tissue removal via clipping. Together, these findings suggest that *O. vulgatum* shows trait‐specific responses to disturbance, with significant reductions in height and leaf area in response to mowing, but only in the absence of herbivory‐like clipping.

**TABLE 1:** Results of ANOVA for *O. vulgatum* parameters. Clipping and mowing were treated as fixed factors and transect as a random factor. When the clipping × mowing interaction was significant the effect of mowing was tested separately for non‐clipped and clipped plants.

Dependent variable	Clipping (1)		Mowing (2)		(1) × (2) interaction		Transect	
	*F*	*p*	*F*	*p*	*F*	*p*	*F*	*p*
(a) Sporophore length	0.121	0.730	0.739	0.379	3.891	0.056	2.842	**0.018**
(b) Number of sporangia	0.210	0.650	0.020	0.888	0.334	0.567	1.366	0.250
(c) Plant height	0.026	0.872	3.410	0.066	7.401	**0.007**	3.497	**0.001**
No clipping			13.297	**0.000**			3.499	**0.003**
Clipping			0.312	0.579			1.244	0.299
(d) Ln (leaf blade area)	0.003	0.957	0.998	0.319	8.062	**0.005**	6.039	**0.000**
No clipping			9.287	**0.003**			6.826	**0.000**
Clipping			1.021	0.317			1.409	0.228

*Note:* In all analyses the significance for intercept *p* < 0.001. Significant effects are bold faced.

## 4 Discussion

### 4.1 Responses to Simulated Herbivory and Mowing in *O. vulgatum*

Our results show that short‐term disturbances can influence the morphology of *O. vulgatum* , although the effects are trait‐specific and dependent on treatment combinations. Importantly, mowing in our experiment was applied only to the surrounding vegetation, not to the fern itself. Therefore, while mowing did not directly damage the ramets, it altered their microenvironment and may have affected growth indirectly through changes in light availability, temperature, and soil moisture. Although such disturbance could be viewed as a release from competition, our findings suggest it may have acted as a physiological stressor under the specific site conditions. The reduction in leaf blade area following mowing may be related to altered microclimatic conditions, including increased soil exposure, evaporation, and reduced humidity, as suggested in previous studies (Brodribb and Holbrook 2004; Chaves et al. 2009). These changes may impair photosynthetic efficiency and reduce available resources for growth. A similar decrease in plant height in mown plots has been observed in other fern species, particularly those sensitive to desiccation (Watkins Jr. et al. 2007).

When considered separately, mowing and simulated herbivory had contrasting effects. Mowing alone significantly reduced plant height and leaf blade area, indicating that the removal of neighboring vegetation adversely affected the performance of *O. vulgatum* .

In contrast, clipping alone, which mimics herbivory, did not significantly affect any of the measured traits. This suggests a degree of tolerance to tissue loss, possibly due to stored resources in the rhizomes and the conservative growth strategy of the species (Johnson‐Groh 1998; McMaster 1994). The lack of a significant negative response to clipping may reflect this species' capacity to sustain aboveground function after partial tissue removal. Nonetheless, it is important to recognize that our treatment was applied only once, and different results may be observed with repeated or chronic herbivory (Belsky 1986; Surendrakumar et al. 2023).

Interestingly, the interaction between mowing and clipping revealed a mitigating effect: negative impacts of mowing on leaf area and height were not observed when plants were also clipped. One possible explanation is that under combined disturbance, resource allocation shifts in a way that buffers the effects of one stressor when the other is present. Alternatively, it may reflect a physiological threshold beyond which additional disturbance does not lead to further suppression. These types of non‐additive effects have been documented in other fern species as well (Watkins Jr. et al. 2007) and highlight the complexity of interpreting multiple interacting disturbances.

### 4.2 Ecological and Conservation Implications

Mowing is a widely used management tool in semi‐natural meadows and has been promoted as a method to maintain open habitats suitable for rare fern species (Müller 2000; Stadnicka‐Futoma and Jaźwa 2020). However, our results suggest that the timing and intensity of mowing are critical. While one‐time mowing did not eliminate *O. vulgatum* individuals from experimental plots, it significantly reduced leaf blade area and plant height in the following season. These traits are closely linked to photosynthetic capacity and spore production, and their reduction may compromise the plant's ability to reproduce and compete in the long term (Schneider et al. 2009). It is worth mentioning that the ability of *O. vulgatum* to propagate clonally through rhizomes may play a key role in buffering the short‐term fluctuations in the performance of the aboveground parts (Camacho and Liston 2001). This aligns with other studies on long‐lived ferns, where sporophyte persistence and vegetative spread help maintain populations despite periodic disturbances (Chung et al. 2012).

Nevertheless, long‐term consequences of repeated disturbance on reproductive success, spore dispersal, and gametophyte recruitment remain poorly understood. Given that *O. vulgatum* has a subterranean gametophyte phase that is difficult to detect and study (Pressel et al. 2016), population dynamics may respond to environmental change on longer time scales than are typically assessed in short‐term field experiments.

In conclusion, our findings suggest that *O. vulgatum* shows a nuanced and trait‐specific response to mowing and simulated herbivory. While short‐term resilience is evident, especially via clonal growth, management practices should consider potential impacts on reproductive output and physiological performance. Further studies are needed to assess the cumulative effects of disturbance regimes over multiple seasons and to understand how they influence both sporophyte and gametophyte phases. Such insights are essential for refining conservation strategies for rare and morphologically simplified fern species in managed grasslands.

### 4.3 Directions for Future Research

Although our study demonstrates the short‐term resilience of *Ophioglossum vulgatum* to disturbance, several key questions remain that warrant systematic investigation to improve both ecological understanding and conservation management.

First, longitudinal demographic studies tracking the fate of both gametophyte and sporophyte generations across multiple years and treatment regimes are essential. Such data would allow testing whether short‐term reductions in photosynthetic area translate into delayed or reduced recruitment and long‐term population decline. Establishing permanent monitoring plots with molecular tagging of sporophyte genotypes could clarify the relative contributions of clonal versus sexual reproduction (Stadnicka‐Futoma et al. 2024).

Second, our findings underscore the need to investigate environmental thresholds influencing reproductive allocation, particularly the interaction between light intensity, soil moisture, and nutrient dynamics. Experimental manipulation of light and water availability could help identify tolerance limits for *O. vulgatum* in response to mowing and drought—a crucial step given climate change projections for Central Europe (Turner and Wright 2023).

Third, there is a strong rationale for comparative studies across populations in different habitat types and management histories, which would clarify the ecological plasticity of the species. Such comparative approaches could incorporate genetic diversity analyses to assess whether resilience and reproductive output are linked to local adaptation or phenotypic plasticity (Chung et al. 2012).

Finally, further studies should also explore the functional traits of trophophores and sporophores under different disturbance regimes using ecophysiological tools such as chlorophyll fluorescence, leaf gas exchange, and isotopic signatures. These metrics would provide insight into the cost‐efficiency of resource allocation strategies and identify physiological constraints under anthropogenic management. Furthermore, research should integrate the potential feedbacks between plant defense traits and environmental pressures, particularly herbivory and disturbance timing (Chavana et al. 2021).

By addressing these directions, future research will not only enrich our ecological understanding of *O. vulgatum* but will also offer practical guidelines for conservation planning in managed meadow ecosystems where rare ferns are part of the biodiversity mosaic. Our study is the first field‐based experimental investigation into how meadow management practices affect resource allocation in *O. vulgatum* , and it provides a valuable starting point for future research using a similar experimental framework.

## Author Contributions

**Natalia Jędrzejczak:** conceptualization (lead), data curation (lead), formal analysis (lead), methodology (lead), resources (lead), visualization (equal), writing – original draft (lead), writing – review and editing (lead). **Paweł Olejniczak:** conceptualization (equal), data curation (equal), methodology (equal), supervision (equal), visualization (equal). **Zbigniew Celka:** conceptualization (equal), methodology (equal), resources (equal), visualization (equal).

## Conflicts of Interest

The authors declare no conflicts of interest.

## Data Availability

The data that support the findings of this study are openly available in “figshare” at http://doi.org/10.6084/m9.figshare.28299494.
